# Protocol for the detection and nutritional management of high-output stomas

**DOI:** 10.1186/s12937-015-0034-z

**Published:** 2015-05-09

**Authors:** Jose J Arenas Villafranca, Cristobal López-Rodríguez, Jimena Abilés, Robin Rivera, Norberto Gándara Adán, Pilar Utrilla Navarro

**Affiliations:** 1 Clinical Medicine and Health Care PhD programme, University of Granada, Granada, Spain; 2 Pharmacy and Nutrition Service, A7, km. 187, Hospital Costa del Sol, Marbella (Málaga), 29603 Spain; 3 Gastroenterology Service, Costa del Sol Hospital, Marbella (Málaga), Spain; 4 General Surgery Service, Costa del Sol Hospital, Marbella (Málaga), Spain; 5 Pharmacology Department, University of Granada, Granada, Spain

**Keywords:** High-output stomas, Hypomagnesaemia, Ileostomy, Readmission, Protocol

## Abstract

**Introduction:**

An issue of recent research interest is excessive stoma output and its relation to electrolyte abnormalities. Some studies have identified this as a precursor of dehydration and renal dysfunction. A prospective study was performed of the complications associated with high-output stomas, to identify their causes, consequences and management.

**Materials and methods:**

This study was carried out by a multidisciplinary team of surgeons, gastroenterologists, nutritionists and hospital pharmacists. High-output stoma (HOS) was defined as output ≥1500 ml for two consecutive days. The subjects included in the study population, 43 patients with a new permanent or temporary stoma, were classified according to the time of HOS onset as early HOS (<3 weeks after initial surgery) or late HOS (≥3 weeks after surgery). Circumstances permitting, a specific protocol for response to HOS was applied. Each patient was followed up until the fourth month after surgery.

**Results:**

Early HOS was observed in 7 (16 %) of the sample population of 43 hospital patients, and late HOS, in 6 of the 37 (16 %) non-early HOS population. By type of stoma, nearly all HOS cases affected ileostomy, rather than colostomy, patients. The patients with early HOS remained in hospital for 18 days post surgery, significantly longer than those with no HOS (12 days). The protocol was applied to the majority of EHOS patients and achieved 100 % effectiveness. 50 % of readmissions were due to altered electrolyte balance. Hypomagnesaemia was observed in 33 % of the late HOS patients.

**Conclusion:**

The protocol developed at our hospital for the detection and management of HOS effectively addresses possible long-term complications arising from poor nutritional status and chronic electrolyte alteration.

## Introduction

The most common causes of intestinal resection in adults are aggressive secondary surgery for vascular and neoplastic disease, and the sequelae of Crohn’s disease with poor pharmacological control [1]. Many of these resections require the construction of a stoma, a surgically-created opening in the abdomen for the discharge of faecal contents, to protect a fragile anastomosis, to manage incontinence or, in the case of certain temporary ostomies, to alleviate symptoms [2].

However, creating a stoma can, in itself, provoke perioperative and postoperative complications, with an estimated incidence of 20–60 % [3, 4]. Local complications that have commonly been reported include peristomal skin damage, infections, parastomal hernia, stenosis, retraction, prolapse and peristomal varices, which have been associated with high rates of morbidity and mortality [5].

An issue of recent research interest is excessive output from the stoma and its relation to electrolyte abnormalities. Some studies have identified this complication as a precursor of dehydration and renal dysfunction, with an estimated incidence of 1–17 %. It is believed to be the cause of 4–43 % of hospital readmissions [3, 6].

High output stomas (HOS) are normally managed by identifying the cause and by treatment consisting of the oral or/and intravenous replacement of water and electrolytes, antisecretory and antidiarrheal medication, nutritional and psychological support [7]. However, there is little information about HOS and most studies reporting rates of complications and mortality following the creation of a stoma do not inform of the incidence, causes and management of HOS.

Hospital readmissions by patients who have undergone colorectal surgery are frequent and costly [8], and HOS are known to delay patient recovery after surgery. Furthermore, HOS constitute a frequent complication in ileostomy patients and are poorly identified; in consequence, the clinical approach adopted is often inadequate. In this prospective study, we examine HOS and identify their possible causes, consequences and management.

## Materials and methods

A prospective, analytical study of a cohort of all patients who underwent surgery resulting in a stoma was carried out. Patients of both sexes, aged 18 years and over, were included. Patients who had undergone previous ostomies were excluded, as were patients who, after surgery, required intensive care for more than 7 days, and those who did not give written informed consent.

Assuming a prevalence of HOS in the overall population of 16 % [7], a sample with 50 patients would achieve a statistical power of over 80 %, for alpha = 0.05. Therefore, 50 patients were recruited after their surgery. All subjects gave their written informed consent. The study was approved by the Hospital’s Ethics Committee, and adhered in all respects and at all times to the Helsinki Declaration.

HOS was defined as ostomy output ≥1500ml for two consecutive days [7, 9, 10]. The patients were classified as early or late according to the time of onset of HOS [7]:

- *Early HOS* (EHOS)*:* Patients who developed HOS in the postoperative phase (<3 weeks after stoma formation), normally before hospital discharge.
- *Late HOS* (LHOS)*:* Patients who developed HOS ≥ 3 weeks after stoma formation.

A multidisciplinary team of surgeons, gastroenterologists, nutritionists and hospital pharmacists was established, and a protocol for response to HOS was designed [11] (Table 1).

**Table 1:** Treatment protocol for high output stomas

Detection and treatment of the underlying cause	
Before initiating pharmacological and nutritional treatment, the underlying cause of the HOS must be detected and treated:	
• Gastrointestinal infections (**after tissue culture assay to rule out*Clostridium difficile*infection**)	
• Related to the medication:	
• Prokinetic indicated drugs: metoclopramide, laxatives, erythromycin, etc.	
• Abrupt withdrawal of corticosteroids	
• Metformin also provokes increased stoma output	
• Bowel obstructions	
• Intra-abdominal sepsis	
• Inflammatory bowel disease	
• Short bowel syndrome	
**Stage I:** *Initial treatment: reduction of fluid and electrolyte loss*	▪ Restrict fluid intake to 500–1000 ml/day. Isotonic drinks are the best option. *Avoid the intake of hypotonic drinks, tea, coffee, alcohol and fruit juices*.
	▪ Perform intravenous hydration.
	▪ Prescribe loperamide 2 mg before breakfast-lunch-dinner and at night.
	▪ Monitoring: strict fluid balance, check body weight daily, perform complete blood analysis including electrolytes (magnesium, calcium, phosphorus, potassium and sodium).
	• Start oral or I/V supplementation of electrolytes if necessary, according to analysis results.
	▪ Start nutritional assessment and treatment *(see below*).
	▪ **Determine B12 levels in patients who are NOT recently operated on**.
	▪ *Determine stoma output at 48–72 h*: if it is resolved, increase oral fluid intake and start serum therapy and the withdrawal of medication.
**Stage II:** *If HOS continues, perform follow up treatment*	▪ Continue the fluid intake restriction and the nutritional monitoring. Start SueroOral intake (2.5 g NaCl, 1.5 g KCl, 2.5 g HCO3Na, 1.5 g sugar and 1 L water) **as the only oral source of fluids** (500–1000 ml/day).
	▪ Increase loperamide dose to 4 mg before breakfast-lunch-dinner and at night (maximum 16 mg/day).
	▪ Start treatment with omeprazole 20 mg/day. If already prescribed, increase to 40 mg/day.
	▪ If fat malabsorption, steatorrhoea, or pruritic bilious output is present, add **cholestyramine** 4 g/12 before breakfast and dinner.
	▪ Continue monitoring and electrolyte supplementation if necessary, as in **Stage I**.
	▪ *If HOS persists after 48–72 h*, initiate **Stage III**.
**Stage III.** *If HOS persists, evaluate treatment and case management*	▪ Supplement with hydro- and lipid-soluble oral vitamins.
	▪ Maintain loperamide and add **codeine** 15**–**60 mg. before breakfast-lunch-dinner. **Contraindicated if the patient has CrCl <15 ml/min**.
	▪ If fat malabsorption persists, increase cholestyramine dose to 4 g before breakfast-lunch-dinner.
	▪ If HOS > 2000 ml **after two weeks**: add octreotide 200 mcg/day for 3–5 days. If no improvement is obtained, suspend this treatment.
	▪ Monitor fluid intake.
**Specific nutritional treatment**	
▪ Avoid fluid intake during meals.	
▪ It may be advisable to temporarily increase the salt content of foods in order to promote fluid reabsorption.	
▪ Little is known about the use of soluble fibre. Insoluble fibre is contraindicated because of the risk of bowel obstruction.	
▪ The effect of antidiarrhoeal microorganisms on HOS is unknown.	

Stoma output was measured for all patients included in the study, carried out over a postoperative follow-up period of four months. Any readmissions needed during this period were recorded.

The descriptive analysis was performed using measures of position and central tendency (median and interquartile range) for quantitative variables and the frequency distribution for qualitative variables. Variables were compared by the chi-square test (or Fisher’s exact test if fewer than 5 observations were expected) for the qualitative variables and the Mann–Whitney *U* test for the quantitative ones. In all analyses, the level of statistical significance was set at p < 0.05.

## Results

Of the 50 patients recruited at the outset, 7 were lost to follow up. Hence, the statistical analysis was performed on a final sample of 43 patients; of these, 63 % were male, with a median age of 66 years (range 58–73).

Colorectal cancer was the most common indication for the formation of a stoma. With respect to the type of surgical intervention, a small majority of cases required a colostomy, and an ileostomy was created in the other cases. Almost half of the interventions were performed as an urgent procedure. Table 2 describes the general characteristics of the study population.

**Table 2:** General characteristics of the population

Baseline characteristics	Total patients (n = 43)
Sex (female/male)	39.5/60.5 %
Age (median, years)	66 (IR 58–73)
Cause of resection	
• *Neoplasia*	74.4 %
• *Inflammatory bowel disease*	14.0 %
• *Benign pathologies*	11.6 %
BMI pre-surgery	23.9 (IR 21.0–28.5)
Variation in BMI at discharge	−0.8 [IR (−2.6)−0.0]
Comorbidities:	
• *Diabetes*	16.3 %
• *Dyslipidaemia*	23.3 %
• *COPD*	4.8 %
• *Hypothyroidism*	4.7 %
• *Hypertension*	39.5 %
Intervention (Urgent/elective)	48.8/51.2 %
Type of stoma (Ileostomy/Colostomy)	46.5/53.5 %
Diagnosis of malnutrition	32.6 %
• *Calorie*	
*○ mild*	4.7 %
• *Protein-Energy*	
* ○ mild*	2.3 %
* ○ moderate*	18.6 %
*○ severe*	7.0 %
Neoadjuvant chemoradiation	
• *Chemotherapy*	13 %
• *Radiotherapy*	6 %
• *Chemoradiotherapy*	12 %
• *None*	69 %
Post-surgery hospital stay (median, days)	13 (IR 9–17)

BMI: Body Mass Index, COPD: Chronic Obstructive Pulmonary Disease, IR: Interquartile range

### High-output stoma

16 % of patients had EHOS and 14 % had LHOS. Table 3 shows the characteristics of the patients with EHOS, LHOS and no HOS. The median period elapsed from surgery to the appearance of EHOS was eight days. The underlying cause of high output was prokinetic agents (14 %), infection (43 %) and unknown (43 %).

**Table 3:** Characteristics of the population with HOS (Early and Late) and the comparison with no HOS population

Baseline characteristics	Patients without early HOS (n = 36)	Patients with early HOS (n = 7)	*p*	Patients without early or late HOS (n = 31)	Patients with late HOS (n = 6)	*p*
Sex (female/male)	44.4/55.6 %	14.3/85.7 %	*p = 0.22*	45/56 %	33.3/66.7 %	*p = 0.68*
Age (median, years)	66 (IR 58–74)	64 (IR 57–68)	*p = 0.44*	66 (IR 59–74)	70 (IR 29–75)	*p = 0.93*
Cause of resection						
• *Neoplasia*	71 %	86 %	*p=0.50*	71 %	83 %	*p=0.47*
• *IBD*	17 %	0 %		16 %	17 %	
• *Benign pathologies*	12 %	14 %		13 %	0 %	
BMI pre-surgery or readmission	23.9 (IR 21.1–27.2)	24.4 (IR 20.7–33.1)	*p = 0.55*	23.9 (IR 20.4–28.5)	23.1 (IR 19.7–29.4)	*p = 0.68*
Variation in BMI at discharge	−0.6 [IR (−2.4)−(0.0)]	−1.5 [IR (−3.4)−(−0.4)]	*p = 0.22*	-	-	*-*
Length of resection (median, cm)	24.7 (IR 18.9–46.8)	24.5 (IR 19.5–40.0)	*p = 0.82*	24.7 (IR 19.5–46.8)	19.5 (IR 16.4–85.0)	*p = 0.72*
Intervention (Urgent/Elective)	50/50 %	43/57 %	*p = 1.00*	48/52 %	50/50 %	*p = 1.00*
Type of stoma (Ileostomy/Colostomy)	36/64 %	100/0 %	*p = 0.002*	29/71 %	83/17 %	*p = 0.02*
Infection post-surgery	31 %	57 %	*p = 0.23*	-	-	*-*
Presence of ileocaecal valve	72 %	71 %	*p = 1.00*	71 %	83 %	*p = 1.00*

IBD: Inflammatory bowel disease, BMI: Body Mass Index, IR: Interquartile range

The protocol was applied to five of the seven EHOS patients. In one case, it was not applied due to spontaneous resolution and in the other, because the patient was lost to follow up. In four of the five to whom it was applied, the HOS was resolved in Phase I protocol, and in the remaining one it was resolved in Phase II (Table 1). These patients received pharmacological support and nutritional recommendations for the control of their HOS, according to the standard protocol [11].

The patients who had postoperative EHOS remained in hospital for 18 days, a significantly longer period than those who did not suffer this complication (12 days) (*U* = 53.00; p = 0.02). With respect to the outcome variables, although the difference was not significant, there was a higher rate of infections among the patients with EHOS (57.1 % vs. 31.4 %; *χ*^2^ = 1.68; p = 0.23). The following variables presented no statistical differences between patients with EHOS and no HOS: age (*U* = 102.00; p = 0.45), sex (*χ*^2^ = 2.23; p = 0.22), reason for surgical intervention (*χ*^2^ = 1.40; p = 0.50), emergency surgery (*χ*^2^ = 0.12, p = 1.00), absence of ileocaecal valve (*χ*^2^ = 0.00; p = 1.00), or malnutrition (*χ*^2^ = 0.01, p = 1.00). Regarding the type of ostomy, all of the EHOS and 83 % of the LHOS cases affected patients with ileostomy (*χ*2 = 9.62; p = 0.002 and *χ*2 = 6.30; p = 0.02, respectively). Thus, the location of the stoma is of evident significance.

The median period from surgery to the appearance of LHOS was 25 days. The protocol was applied to 33 % of LHOS patients (all resolved in Phase I). The rest did not receive protocol care because they were readmitted for non-general surgery services (Internal Medicine and Emergency). The underlying cause of the LHOS was not detected in any of these cases. 50 % of these patients were given pharmacological support and nutritional recommendations by clinicians to control the high output at discharge. However, 17 % were given erroneous recommendations based on increasing fluid intake, and no pharmacological control was recommended to control the high output.

### Readmission

28 % of the stoma patients were subsequently readmitted to hospital, with a mean weight loss from discharge to readmission of 5.2 ± 2.3 kg. In 50 % of the patients who were readmitted for LHOS, the principal cause was altered electrolyte balance and dehydration. Electrolyte abnormalities were detected in 100 % of these patients on readmission. Magnesium levels were monitored in three of the six LHOS, and hypomagnesaemia was observed in two patients.

No differences were found between LHOS and no HOS patients regarding the following variables related to readmission: age (*U* = 91.00; p = 0.93), gender (*χ*^2^ = 0.29; p = 0.68), cause of surgical intervention (*χ*^2^ = 0.88; p = 0.47), urgent surgery (*χ*^2^ = 0.01; p = 1.00), absence of ileocaecal valve (*χ*^2^ = 0.39; p = 1.00), neoadjuvant chemotherapy (*χ*^2^ = 3.18; p = 0.11), neoadjuvant radiotherapy (*χ*^2^ = 1.41; p = 0.27), BMI at discharge *U* = 75.00, p = 0.68), or malnutrition (*χ*^2^ = 1.22, p = 0.58).

## Discussion

The need to construct a stoma often arises in clinical practice [3]. According to statistical records, 1.5 per thousand of the Spanish population (i.e., 60,000 patients) live with a stoma. Taking the age factor into account, the incidence of stoma corresponds to over 3 per thousand of the adult population in Spain. In other Western countries, figures of 2–4 per thousand of the adult population [12] have been reported.

In our study population, colorectal cancer was the most common reason for a stoma being needed (65.1 % of cases); this finding is consistent with results reported elsewhere [3, 5].

During the first days after the construction of an ostomy, faecal effluent usually increases, but it decreases rapidly following intestinal adaptation [13]. When this adaptation fails or is prolonged, patients must face the challenge of controlling large losses of fluid that can lead to a state of chronic dehydration [14]. When these losses occur, ostomy patients present major deficits of water, sodium and magnesium [15] and can also suffer malnutrition and long-term weight loss. This event is known as HOS, and among stoma patients our study revealed a prevalence of EHOS of 16 %, which is consistent with Baker et al. [7]. These same authors identified the underlying cause of HOS in 50 % of cases, and noted the presence of short bowel syndrome, treated by medication, and subsequent intestinal obstruction. In our study, the underlying cause was identified in 57 % of the cases, with drug treatment or the presence of infection being mainly responsible for the occurrence of EHOS. None of the patients presented short bowel syndrome.

It has been reported that HOS tend to occur more frequently after emergency surgery [16], but we observed no such association. There was a relationship between infections and the presence of EHOS, but it was not statistically significant [17]. On the other hand, there were significant differences according to the type of ostomy, for both EHOS and LHOS when an ileostomy was performed. This finding corroborates an earlier meta-analysis of all complications arising from ostomies – classifying the latter as colostomy or ileostomy – which concluded that only HOS presented significant differences, being more common following an ileostomy [18].

We compared HOS and non-HOS patients, and found no significant differences in the universal defining variables, or with regard to the causes that led to the surgical intervention. Despite the importance of the presence of the ileocaecal valve in retaining stoma output and that of the secretion of hormones favouring intestinal adaptation after surgical resection [1], we observed no differences with regard to the development of high output. Neither were there significant differences between patients who received prior chemotherapy or radiotherapy, as have been described by Hayden et al. [8]. However, we did find significant differences with respect to the longer hospital stay required by patients with HOS, thus confirming the results of Harris et al. [3].

With respect to the outcome variables, patients with HOS presented a sharper decrease in BMI at hospital discharge and a higher rate of infections. The latter has been reported to be one of the risk factors associated with the occurrence of HOS [19]. There were no significant alterations in the electrolyte profile in patients with EHOS, and no hypomagnesaemia was detected at first hospital admission in any patient, in contrast to the 45 % of hypomagnesaemias reported by Baker [7].

An interesting finding is that EHOS appears, on average, eight days after resection. This highlights the importance of instructing the patient in this respect before hospital discharge, especially in cases of early discharge when the condition has evolved favourably. Several papers have commented on the importance of these patients being instructed and informed by a multidisciplinary nutrition team including pharmacists, nutritionists, nurses and surgeons [17, 20, 21], while the NICE guidelines for ostomy patients state that they should be examined every 2–3 months by a nutritional team after the surgical intervention [22].

The early examination and treatment protocol developed for these patients (Table 1) was applied to the majority of patients with HOS and achieved 100 % effectiveness. In clinical practice, it is important to test for *Clostridium difficile* in order to detect the underlying cause of high output, as an increasing number of cases of diarrhoea have been associated with this agent during hospitalization, which affects 3–10 patients per 1000 hospitalizations [23]. Moreover, studies have reported cases of colonisations in the small intestine causing high output in patients with ileostomy [24, 25]. As part of the above protocol, nutritional and pharmacological recommendations were made to 83 % of EHOS patients and to 50 % of the LHOS patients, who were thus enabled to achieve self-management of stoma output. This outcome represents a significant improvement in our hospital, where a previous retrospective study had noted a complete absence of recommendations to patients on hospital discharge [26].

The proportion of ostomy patients who were readmitted with altered electrolyte balance and dehydration because of LHOS was 50 %, much higher than the 5.5 % reported by Baker [7], but similar to that reported in other studies, which have recorded 20–43 % of readmissions due to dehydration related to fluid losses through the stoma [8]. Ileostomy is considered a risk factor for kidney failure [27]. In our study, 50 % of kidney failures were secondary to stoma losses, which is in accordance with the 30–71 % of the patients with HOS reported elsewhere to have developed acute kidney failure secondary to dehydration [8]. In our study, the cause of LHOS was not determined in any patient, nor were tests applied to detect the presence of *Clostridium difficile*. The protocol was only applied in 33 % of LHOS cases, all of whom were patients admitted by the Surgery Service, where personnel had been previously instructed with respect to HOS and the recommended treatment. Among these 33 %, the follow up rate was 100 %. Moreover, the existence of this protocol enabled nutritional and pharmacological recommendations to be made on hospital discharge to 50 % of the patients admitted for surgery or nephrology. However, 17 % of stoma patients received recommendations contrary to those contained in the literature on the subject and were given no pharmacological treatment to control the stoma output. A study published in 2012 examined the rate of readmissions due to dehydration among patients with ileostomy, comparing two periods, one of which included the implementation of a perioperative protocol of patient education on how HOS should be managed; this study reported a significant reduction in the rate of readmissions, although there were no significant differences in the duration of hospital stay [28]. In our opinion, HOS management should be extended throughout the medical service in order to improve the care provided for ostomy patients care during readmissions.

Decreased levels of Mg in plasma are very common following an intestinal resection, due to the consequent reduction in absorption surface area and to chelation with fatty acids [29, 30]. This condition can affect up to 45 % of patients with HOS due to hypoaldosteronism secondary to surgery and often appears in long-term HOS [7]. Unfortunately, it is not often monitored as routine clinical practice in Spain. Another common consequence is the appearance of electrolyte alterations such as hypokalaemia and hypocalcaemia, both of which are refractory to treatment until the Mg deficit is corrected. Thus, some of our patients presented simultaneous deficits of Mg, Na, K and P.

A limitation of this study is the small sample size, which means that the conclusions drawn cannot be considered definitive.

## Conclusions

A review of stoma output should be included in standard clinical practice, in view of the comorbidity of this condition, which is demonstrated in the present study. The protocol developed at our hospital for the detection and management of HOS has proved to be effective in addressing possible long-term complications arising from poor nutritional status and chronic electrolyte alterations, which may provoke severe consequences among the patients affected.
